# Auditory objects in working memory include task-irrelevant features

**DOI:** 10.1038/s41598-024-72177-6

**Published:** 2024-09-11

**Authors:** Cora Fischer, Carina Nolting, Flavia Schneider, Christoph Bledowski, Jochen Kaiser

**Affiliations:** Institute of Medical Psychology, Faculty of Medicine, Goethe University Frankfurt am Main, Heinrich-Hoffmann-Str. 10, 60528 Frankfurt am Main, Germany

**Keywords:** Auditory working memory, Object-based attention, Irrelevant feature, Irrelevant change effect, Frequency, Location, Human behaviour, Attention

## Abstract

Object-based attention operates both in perception and visual working memory. While the efficient perception of auditory stimuli also requires the formation of auditory objects, little is known about their role in auditory working memory (AWM). To investigate whether attention to one object feature in AWM leads to the involuntary maintenance of another, task-irrelevant feature, we conducted four experiments. Stimuli were abstract sounds that differed on the dimensions frequency and location, only one of which was task-relevant in each experiment. The first two experiments required a match–nonmatch decision about a probe sound whose irrelevant feature value could either be identical to or differ from the memorized stimulus. Matches on the relevant dimension were detected more accurately when the irrelevant feature matched as well, whereas for nonmatches on the relevant dimension, performance was better for irrelevant feature nonmatches. Signal-detection analysis showed that changes of irrelevant frequency reduced the sensitivity for sound location. Two further experiments used continuous report tasks. When location was the target feature, changes of irrelevant sound frequency had an impact on both recall error and adjustment time. Irrelevant location changes affected adjustment time only. In summary, object-based attention led to a concurrent maintenance of task-irrelevant sound features in AWM.

## Introduction

Objects form key elements of perception and attentional selection^1^. In particular, attending to one object feature can lead to a processing benefit for other features of the same object compared with features of different objects. This phenomenon, which is known as object-based attention^2^, is reflected by more accurate or faster responses^3,4^ and by increased hemodynamic activity evoked by same-object features^5–7^. In working memory, object-based attention leads to faster responses and increased hemodynamic activity for memorized features within the same object^8,9^. Encoding a task-relevant feature can give rise also to an involuntary encoding of task-irrelevant features. However, the extent and the conditions under which irrelevant features influence the memory recall of relevant features are a matter of debate^10–13^.

While most research on object-based processing has been performed in the visual modality, objects are thought to play an important role also in auditory perception. The efficient processing of auditory signals requires the formation and memory representation of auditory objects^14,15^. Similar to the visual modality, the object-based model of auditory attention states that attention can be allocated to selected objects^16,17^ and that attentional selection of one particular object feature enhances the processing of other features of the same object^18^.

In studies showing that attention allocation to one object feature facilitates the processing of unattended same-object features^3,4,8,18^, the unattended feature could also be tested on some occasions. Thus, the unattended feature was never completely task-irrelevant throughout one experiment. However, the object-based processing benefit should also apply to features that are never task-relevant. This can be probed with eye-movement analyses, showing for example that gaze shifts occur towards the task-irrelevant spatial position of a selected item^19^. Alternatively, implicit measures have been proposed like the irrelevant change effect, which describes the disruption of memory performance for the relevant feature caused by changes of the irrelevant feature at memory test^12^. In the visual domain, irrelevant change effects have been observed for mismatches of different stimulus features including location, color or shape^20–22^. In the auditory modality, Maybery et al.^23^ assessed working memory for sequences of either spoken letters or abstract sounds presented from different loudspeakers. Spatial memory performance was reduced when the task-irrelevant spoken letter was changed at test, whereas verbal memory performance was unaffected by location changes. A further AWM study using musical sounds found that changes of task-irrelevant pitch or timbre affected location recall, whereas irrelevant location changes did not affect either pitch or timbre recall^24^. Apparently, irrelevant frequency-related changes played a bigger role than irrelevant spatial changes in AWM.

The present study investigated the role of task-irrelevant stimulus features in AWM. For this aim we asked participants to encode explicitly one feature of each of two abstract noise stimuli, while other stimulus features were task-irrelevant. After a short delay, subjects were instructed about which stimulus had to be memorized. At test, a probe sound was presented consisting of both the task-relevant and the task-irrelevant feature. This procedure was similar to recent neuroimaging AWM studies^25–27^ and allowed us to test whether working memory performance regarding the task-relevant stimulus feature was affected by an additional, involuntary processing of the task-irrelevant stimulus feature. In the first two experiments, participants had to decide if a probe sound matched the memorized sound after a retention phase. However, it is conceivable that information about irrelevant features is used inadvertently when participants are asked to compare the identity of probe and memorized stimuli.

To test whether irrelevant features impact working memory performance even when the active recall of a single relevant feature value is required instead of a comparison with a test sound, we conducted two additional experiments. Both experiments used the same sample stimuli and retro-cue manipulation. However, participants now had to adjust the relevant feature of a test stimulus freely by moving a computer mouse until it matched the memorized value. Again, the irrelevant feature was either identical or differed between retro-cued target and test stimulus. Assuming that irrelevant features are co-activated by attentional selection of relevant features of a sound object, we expected that variations of the irrelevant feature would affect both recall error and adjustment times. In addition, we compared the irrelevant change effect between trials with a smaller versus larger difference of the irrelevant feature between target and test stimuli. We expected that larger differences would have a stronger effect on performance than smaller differences.

## Experiment 1

The first experiment used a retro-cue delayed match-to-sample task with sound location as the task-relevant feature and frequency as the task-irrelevant feature. We assessed accuracy and reaction time for probe stimuli with matching versus nonmatching relevant and irrelevant features.

### Methods

#### Participants

To estimate the sample size, we based a power analysis on the means and standard errors reported in Table 2 of Delogu et al.^24^, whose paradigm was to some extent comparable with the present match–nonmatch task. For accuracy of match trials in their study, effect size amounted to Cohen’s d = 1.0; for reaction times, effect sizes varied between d = 0.59 and 0.82 depending on the contrast. For an alpha level of 0.05 and a power of 0.80, this would result in sample sizes of N = 8 (for accuracy) and N = 20 (for reaction time). Consequently, we chose a sample size of N = 20 for all four experiments.

Twenty healthy young adults (14 females, mean age 27.3, standard deviation (SD) = 5.8 years) participated in the first experiment. They were either university students or held academic degrees. They had normal or corrected-to-normal vision and normal hearing abilities. Written informed consent was obtained from all observers. They were naive to the purpose of the experiment and received a remuneration of €10 per hour. Approval by the ethics committee of the Goethe University medical faculty was obtained for all four experiments (No. 390/16). We confirm that all experiments were performed in accordance with relevant guidelines and regulations.

#### Stimuli

**Fig. 1:**
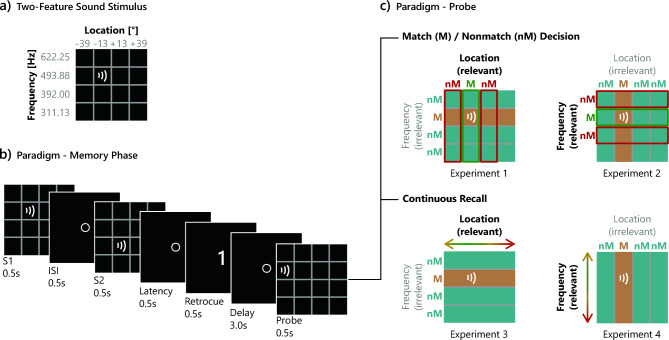
Experimental design for the four experiments. (**a**) Two-feature sound stimuli: sample sounds were generated as combinations of two feature dimensions, central frequency (e.g., 493.88 Hz) and perceived location (e.g., 13° left of the frontal midline). (**b**) Paradigm—memory phase: in each trial, two sounds (S1 and S2) were presented sequentially. Note that stimuli were presented only acoustically, the grids shown here only serve illustrative purposes. During presentation, the fixation circle remained on the screen. A visual retro-cue indicated the target stimulus that had to be maintained in AWM during the delay period. (**c**) Paradigm—probe: probe types and recall paradigms are shown for the four experiments: In Experiments 1 and 2 (top row), participants had to judge whether the task-relevant feature of a probe sound matched the feature of the target memory sound (match, M) or not (nonmatch, nM). Green and red frames indicate sound feature combinations that could serve as possible match or nonmatch probes, respectively. In all experiments, the task-irrelevant feature of the probe sound could either match the irrelevant feature of the target sound or not. Brown and turquoise rows or columns indicate sound feature combinations of probes with matching versus nonmatching feature values on the irrelevant dimension, respectively. In Experiments 3 and 4 (bottom row), a test sound was presented with a randomly chosen value for the task-relevant dimension that had to be adjusted until it matched the memorized target sound.

The stimuli were two-dimensional feature combinations of a spatial location and a central frequency. All sounds had a duration of 500 ms with rise and fall times of ~ 50 ms. The presented sounds consisted of filtered noise with central frequencies of 311.13, 392.0, 493.88 or 622.25 Hz with a bandwidth of 1/6 octave. The spatial stimulus feature was generated by introducing an interaural time difference (ITD), i.e., a sound onset delay between the left and right headphone of ± 0.12 ms or ± 0.35 ms, corresponding roughly to lateralizations of 13° or 39° from center to the left or to the right, respectively. We created spatial differences via ITD manipulations to interfere as little as possible with the sound frequency patterns. Even though ITD variations lead to the impression of sounds lateralized within one’s head rather than in the environment, we preferred to use ITD manipulations because other methods like head-related transfer functions, which model the frequency-specific filtering properties of the head and outer ear, would have included frequency-specific modifications of the sound patterns. The pool of sample stimuli thus consisted of 16 (4 × 4) different combinations of frequency and location (Fig. 1a). Probe sounds matched the location of the target sample sound in half of the trials. In the other half of trials, nonmatching probe sounds were presented that could differ from the samples at a perceived distance of either ± 35° or ± 45°. Non-matching probes thus never appeared at a possible location for a target. These values were chosen on the basis of pilot work to create trials with more or less difficult comparisons. The exact ITDs are shown in Table 1. Furthermore, in half of the trials, the frequency of the probe sound either matched the target frequency or had one of the two frequencies that had not been presented in the trial yet. Stimulus construction and timing were controlled with MATLAB R2012b (The MathWorks) and the Psychophysics Toolbox^28^. Stimuli were processed with an external soundcard (Fireface UC, 192 kHz sampling rate, RME) and presented via headphones (AKG K271) at a comfortable intensity (~ 85–95 dB SPL).

**Table 1:** Perceived locations relative to the frontal midline and corresponding ITDs (in parentheses) for the sample sounds and the nonidentical probe stimuli.

Sample sounds	Nonmatching probes			
	− 35°	− 45°	+ 35°	+ 45°
13° (0.12 ms)	− 22° (− 0.20 ms)	− 32° (− 0.29 ms)	48° (0.42 ms)	58° (0.49 ms)
39° (0.35 ms)	4° (0.04 ms)	− 6° (− 0.06 ms)	74° (0.60 ms)	84° (0.65 ms)

A minus denotes the opposite side of the frontal midline. For example, for the sample stimulus at 13° on the right of the midline, probes with a difference of − 35°/− 45° were presented on the left of the midline.

#### Procedure

The experiment took place in a dimly lit room. Participants were seated in front of an LCD monitor at a distance of about 50 cm. The trial structure is illustrated in Fig. 1b. After the participants had been instructed about the task and the experimental procedure, they performed a practice block with 20 trials. The actual experiment consisted of 512 trials divided into 8 blocks with 64 trials each. The participants could take a break after each block and started the next block by themselves via mouse click.

Trials began with the presentation of a white fixation circle (diameter: 20 pixels) at the center of the monitor and the sequential presentation of the two sample sounds. They were separated by an interstimulus interval of 500 ms. The sample sounds were characterized by a combination of the four perceived locations and four frequencies described in the section above. They always differed from one another both in location and frequency. After a brief delay of 500 ms, the second sample stimulus was followed by a visual retro-cue (the digit “1” or “2”), indicating the serial position of the target sound whose location had to be memorized across the 3-s delay phase. Whether the first or second sound was the target stimulus was balanced. Then the probe stimulus was presented and participants had to indicate whether its location matched the location of the target sound or not by pressing the left or the right key of a computer mouse, respectively (Fig. 1c). In half of the trials, the location of the probe sound matched the target, while in the other half the location differed by either ± 35 or ± 45° (balanced as well). In the trials with a location nonmatch, smaller and larger location differences were presented equally often. The irrelevant feature frequency was either identical or differed between target and probe sound in each half of the match and nonmatch trials, respectively. Note that the factors target location, target frequency, location variation and frequency variation were completely counterbalanced. Feedback was given immediately after the response, or at the latest 1.5 s after probe stimulus presentation, corresponding to the upper time limit for the response. A white tick mark or a white cross were presented for correct or incorrect responses, respectively. During the 1.5-s inter-trial interval, the fixation circle turned gray.

#### Data analysis

Accuracy (correct response rate) and reaction times for correct trials were analyzed in separate 2 × 2 repeated-measures analyses of variance (ANOVA) with the factors Location Variation (match versus nonmatch) and Frequency Variation (match versus nonmatch). To distinguish between accuracy and response bias, we also determined signal detection theory-based indicators of sensitivity (d′) and criterion (c) based on the normalized hit and false alarm rates for nonmatches of location. Here c > 0 indicated a bias towards “match” responses. These parameters were calculated separately for frequency matches and nonmatches. In addition, we conducted an exploratory analysis to assess the effect of the magnitude of irrelevant feature change on the irrelevant change effect. Here only those trials with a change of the irrelevant feature (half of the total number of trials) were entered into the analysis. We contrasted trials with a smaller and a larger difference of the irrelevant feature between target and probe sounds to test whether the irrelevant change effect scaled with the magnitude of the difference on the irrelevant feature dimension. We performed repeated-measures ANOVAs with the within-subject factors Frequency Change Magnitude (two levels: smaller versus larger differences between sample and probe frequency) and Location Variation (match versus nonmatch trials) for trials with frequency changes. This was done for both accuracy and reaction time.

Data, study materials and analysis code for all four experiments are available upon request from the authors.

### Results

**Fig. 2:**
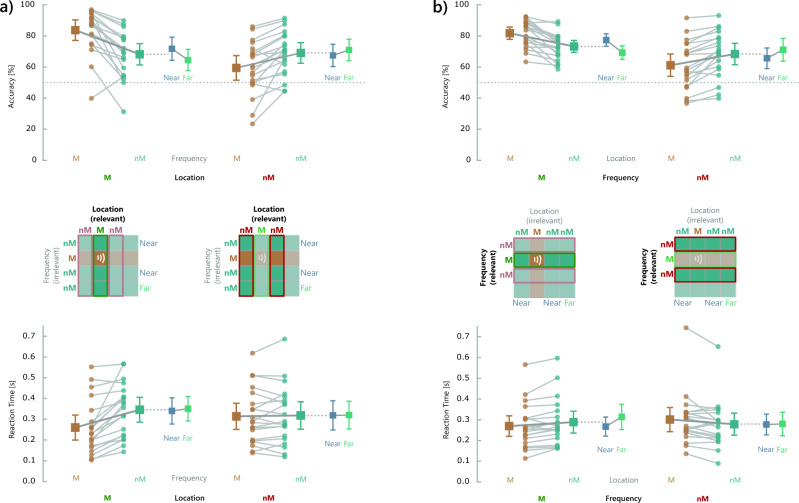
(**a**) Results of Experiment 1 with location as the task-relevant feature and frequency as the irrelevant feature. The upper panel shows the accuracy [% correct responses] and the lower panel shows reaction times [s] for location match (M) and nonmatch (nM) trials as a function of variations of the irrelevant feature. Data for trials with identical values of the irrelevant feature for target and probe stimuli are shown in brown, whereas trials with a change of the irrelevant feature are shown in turquoise. Colored dots and light gray connecting lines indicate the values of individual participants. Larger squares represent the condition mean with vertical bars indicating the standard error. Smaller squares on the right of each graph show mean and standard error for trials with a smaller difference of the irrelevant feature (“near”, shown in blue) and for trials with a larger difference of the irrelevant feature between sample and test stimuli (“far”, shown in green). (**b**) Results of Experiment 2 with frequency as the task-relevant feature and location as the irrelevant feature.

**Fig. 3:**
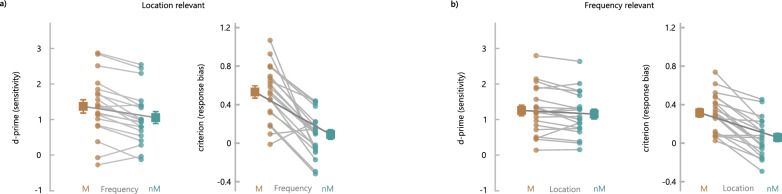
Signal detection parameters for (**a**) Experiment 1 and (**b**) Experiment 2. The left graphs of each part show the sensitivity (d′) and the right graphs depict the criterion (**c**). Data for trials with identical values of the irrelevant feature for target and probe stimuli are shown in brown, whereas trials with a change of the irrelevant feature are shown in turquoise. Colored dots and light gray connecting lines indicate the values of individual participants. Larger squares represent the condition mean with vertical bars indicating the standard error.

The ANOVA for accuracy yielded significant main effects of both Location Variation (p = 0.002) and Frequency Variation (p = 0.005) and an interaction between both factors (p < 0.001) (Fig. 2a, upper panel). Details of the ANOVA results are provided in Supplementary Table S1, and means and SD for the different conditions are given in Supplementary Table S2. Accuracy for match trials with identical location for sample and probe sounds was higher when both stimuli also matched on the task-irrelevant feature frequency than when there was a frequency nonmatch (t(19) = 6.32, p < 0.001). In contrast, for location nonmatch trials, accuracy was lower when task-irrelevant frequency of sample and probe was identical than when both stimuli differed also in frequency (t(19) = 3.91, p = 0.002). Signal detection analysis (Fig. 3a) showed that the sensitivity of location recall was reduced for changes of the irrelevant feature frequency between target and probe sounds (frequency match: d′ = 1.37 (SD: 0.84), frequency nonmatch: d′ = 1.05 (SD: 0.74), t(19) = 4.99, p < 0.001). In addition, for frequency matches, there was an increased bias towards ‘match’ responses compared with frequency change, while there was no bias for frequency nonmatches (frequency match: c = 0.43 (SD: 0.29), frequency nonmatch: c = -0.02 (SD: 0.23), t(19) = 6.71, p < 0.001).

In addition, the magnitude of change of the irrelevant feature was associated with the strength of the irrelevant change effect (Fig. 2a, upper panel). The ANOVAs for correct response rate yielded neither main effects of Frequency Change Magnitude (p = 0.272) nor Location Variation (p = 0.768), but an interaction between both factors (p = 0.002) (Supplementary Table S1). For location match trials, smaller frequency changes (“near”) were associated with a higher accuracy than larger changes (“far”) (t(19) = 3.59, p < 0.001, Cohen’s d = 0.802). Conversely, for location nonmatch trials, smaller frequency changes tended to lead to a lower accuracy than larger frequency change (t(19) = 1.48, p = 0.078, Cohen’s d = 0.33). Means and SDs for the different conditions are given in Supplementary Table S3.

For reaction time (Fig. 2a, lower panel), we found a main effect of the factor Frequency Variation (p < 0.001) and an interaction between both factors (p < 0.001), but no main effect of Location Variation (p = 0.297) (Supplementary Table S1). The interaction was accounted for by the differential effect of the irrelevant feature on location match versus nonmatch trials. For location match trials, participants responded faster when there was a frequency match compared with frequency nonmatch (t(19) = 6.51, p < 0.001). In contrast, the irrelevant feature did not affect reaction time to location nonmatch trials (t(19) = 0.32, p = 0.751). Means and SDs for the different conditions are provided in Supplementary Table S2. Assessing the effects of change magnitude of irrelevant frequency, for reaction time there was a trend for a main effect of Location Variation (p = 0.083), but neither a main effect of Frequency Change Magnitude (p = 0.685) nor an interaction between both factors (p = 0.436) (Fig. 2a, lower panel, and Supplementary Table S1).

### Summary

Changes of task-irrelevant frequency affected memory performance, and this effect scaled with the magnitude of difference of the irrelevant feature. Signal detection analysis showed that changes of the irrelevant feature reduced the sensitivity of location recall in addition to affecting the criterion. This supported the notion that the irrelevant feature was not deleted from memory, but rather attending to task-relevant location led to an automatic processing, including both encoding and memorization, of task-irrelevant frequency in AWM.

## Experiment 2

After Experiment 1 had shown effects of task-irrelevant frequency on working memory for sound locations, Experiment 2 investigated whether task-irrelevant location would affect working memory for sound frequency in a similar way. We used the same stimuli and experimental design, but reversed the roles of both task features. In Experiment 2, participants had to memorize the frequency of the retro-cued sample sound and compare it with the frequency of a probe sound, whereas sound location was task-irrelevant.

### Methods

#### Participants

Twenty healthy young adults (14 females, mean age 21.9, SD = 1.83 years) participated in Experiment 2. This group fulfilled the same criteria as in Experiment 1 but did not overlap with the participants from the first experiment. Written informed consent was obtained from all observers. They were naive to the purpose of the study and received a remuneration of €10 per hour.

#### Stimuli

The same sample stimuli were used as in Experiment 1. Probe sounds matched the frequency of the target sample sound in half of the trials. In the other half of trials, nonmatching probe sounds were presented that could differ from the samples either by ± 0.038 log(10) Hz or by ± 0.05 log(10) Hz. These values were chosen on the basis of pilot work to create trials with more or less difficult comparisons. The exact frequency values are shown in Table 2.

**Table 2:** Central frequencies for the sample sounds and the nonidentical probe stimuli.

Sample sounds	Nonmatching probes			
	− 0.05 log(10)	− 0.038 log(10)	+ 0.038 log(10)	+ 0.05 log(10)
311.13 Hz	277.18 Hz	285.30 Hz	339.29 Hz	349.23 Hz
392.00 Hz	349.23 Hz	359.46 Hz	427.47 Hz	440.00 Hz
493.88 Hz	440.00 Hz	452.89 Hz	538.58 Hz	554.37 Hz
622.25 Hz	554.37 Hz	570.61 Hz	678.57 Hz	698.46 Hz

#### Procedure

The experimental settings and the procedure were almost identical to Experiment 1. The main difference was that sound frequency was now the task-relevant feature that had to be compared between target sample and probe stimulus. Again, the frequency of the probe sound matched the target in half of the trials, whereas in the other half the frequency differed by either ± 0.038 log(10) Hz or by ± 0.05 log(10) Hz. Smaller and larger frequency differences were presented equally often. Furthermore, in half of the trials, the location of the probe sound either matched the target location or had one of the two locations that had not been presented in the trial yet. Again the factors target position, frequency, location, frequency variation and location variation were completely counterbalanced.

#### Data analysis

The data analysis followed the same principles as in Experiment 1.

### Results

For accuracy, we found a main effect of Frequency Variation (p < 0.001) with better performance for match than nonmatch trials, but no main effect of Location Variation (p = 0.396) (Fig. 2b, upper panel, and Supplementary Tables S4 and S5). There was a significant interaction between both factors (p < 0.001). Similar to Experiment 1, accuracy for trials with identical frequency for sample and probe sounds was higher when both stimuli also matched on the task-irrelevant feature location than when there was a location nonmatch (t(19) = 6.52, p < 0.001). In contrast, for frequency nonmatch trials, accuracy was lower when task-irrelevant location of sample and probe was identical than when both stimuli also differed in location (t(19) = 5.53, p < 0.001). Signal detection analysis (Fig. 3b) revealed a trend for reduced sensitivity of frequency recall for changes of the irrelevant feature location (location match: d′ = 1.26 (SD: 0.66), location nonmatch: d′ = 1.16 (SD: 0.61), t(19) = 2.05, p = 0.055). As in Experiment 1, matches of the irrelevant feature led to an increased bias towards ‘match’ responses compared with irrelevant feature changes (location match: c = 0.32 (SD: 0.20), location nonmatch: c = 0.06 (SD: 0.20), t(19) = 7.47, p < 0.001).

As in Experiment 1, we assessed whether the magnitude of change of the irrelevant feature was associated with the strength of the irrelevant change effect (Fig. 2b, upper panel, and Supplementary Tables S4 and S6). The ANOVAs for correct response rate yielded neither main effects of Location Change Magnitude (p = 0.236) nor Frequency Variation (p = 0.123), but an interaction between both factors (p < 0.001). For frequency match trials, smaller location changes (“near”) were associated with a higher accuracy than larger changes (“far”) (t(19) = 5.51, p < 0.001, Cohen’s d = 1.23). Conversely, for frequency nonmatch trials, smaller location changes led to a lower accuracy than larger location changes (t(19) = 4.04, p < 0.001, Cohen’s d = 0.903).

For reaction time, we found no main effects of Frequency Variation (p = 0.462) or Location Variation (p = 0.730), but an interaction between both factors (p < 0.001) (Fig. 2b, lower panel, and Supplementary Tables S4 and S5). The interaction was accounted for by the differential effect of the irrelevant feature on frequency match versus nonmatch trials. For frequency match trials, participants tended to respond faster when there was a location match than for location nonmatches (t(19) = 2.63, p = 0.061). The opposite effect was found for frequency nonmatch trials, where reaction times were slower when there was a location match than for location nonmatches (t(19) = 3.11, p = 0.021).

Exploring the effects of irrelevant change magnitude, we found a main effect of Location Change Magnitude (p = 0.011), but not of Frequency Variation (p = 0.434), and an interaction between both variables (p < 0.001) (Fig. 2b, lower panel, and Supplementary Table S4). For frequency match trials, smaller location changes were associated with a shorter reaction time than larger changes (correct response rate for small location change: 0.267 s, SD = 0.098 s, for large location change: 0.314 s, SD = 0.129 s, t(19) = 4.13, p < 0.001, Cohen’s d = 0.924). In contrast, for frequency nonmatch trials, there was no difference between smaller versus larger location changes (t(19) = 0.27, p = 0.61).

### Summary

Performance on the frequency working memory task was modulated by variations of the irrelevant feature sound location. As in Experiment 1, the effects of irrelevant feature change scaled with its magnitude. This supported the notion that attention to task-relevant frequency led to a memory representation of task-irrelevant location of the target sound in AWM. The analysis of signal-detection parameters showed that the irrelevant location changes strongly affected response bias whereas there was merely a statistical trend for reduced sensitivity.

## Experiment 3

Experiments 3 and 4 investigated whether irrelevant features effects on working memory performance generalize from a match-to-sample design to a task requiring the active recall of a single relevant feature value. While we used the same sample stimuli and retro-cue manipulation as in the first two experiments, here participants had to adjust the relevant feature of a test stimulus to match the value of the memorized stimulus. Matching a test stimulus to a memorized sound that both consist of relevant and irrelevant features might encourage the inadvertent use of information about the irrelevant feature for decision making during memory recognition, especially in the case of uncertainty about the relevant feature. To investigate whether irrelevant feature effects were partly attributable to the task design and whether they also apply in situations with an active reproduction of a relevant feature, Experiments 3 and 4 required the continuous recall of the relevant feature value. We anticipated that irrelevant change effects might be less pronounced than in the match–nonmatch tasks, as it might be easier to separate relevant and irrelevant features in the active recall task. However, assuming that sounds are represented in AWM as bound objects, we still expected that irrelevant feature changes would affect active recall performance.

### Methods

#### Participants

Twenty healthy young adults (14 females, mean age 23.1, SD = 3.58 years) participated in Experiment 3. They fulfilled the same criteria as in Experiment 1 or 2 and had not participated in any of the previous experiments. Written informed consent was obtained from all observers. They were naive to the purpose of the experiment and received a remuneration of €12 per hour.

#### Stimuli

The same sample stimuli were used as in Experiment 1 and 2. The location of the test sounds was drawn randomly from the range of perceived lateralization angles of ± 90° from the frontal midline. In contrast, the frequency of the test sound matched the target frequency in half of the trials, whereas in the other half of trials, it had one of the two frequencies that had not been presented in the trial yet.

#### Procedure

The experimental settings and the procedure were almost identical to Experiments 1 and 2. Again, 512 trials were divided into 8 blocks with 64 trials each. The main difference was that instead of making a match–nonmatch decision, participants had to perform a continuous recall task. After the 3-s delay phase, a probe tone was presented. The probe sound lasted 500 ms and was continuously repeated either until a response was given or the 20-s response window ended. Participants adjusted the target feature location by moving a computer mouse to the left or right and entered their response via left mouse button press. The continuous response space for location ranged from 90° on the left to 90° on the right with a maximum interaural time difference of 0.68 ms. Participants received feedback about their recall accuracy after the response, when the fixation circle took on a color between green and red for 500 ms to indicate the error magnitude.

#### Data analysis

As a measure of recall error, we calculated the mean of the absolute value of the deviation from the target feature value for each participant. This was compared between trials with and without a change of the irrelevant dimension by repeated-measures *t*-tests. As we had specific hypotheses, one-tailed *t*-tests were used. Analogous to the first two experiments, we also assessed adjustment times. They were determined as the time from the onset of the probe stimulus to the button press and compared between trial types. In a further analysis, we focused on trials with nonmatches on the irrelevant feature dimension and compared trials with a smaller and a larger difference of the irrelevant feature between target and probe sounds. Again, this was done for both recall error and adjustment time.

### Results

**Fig. 4:**
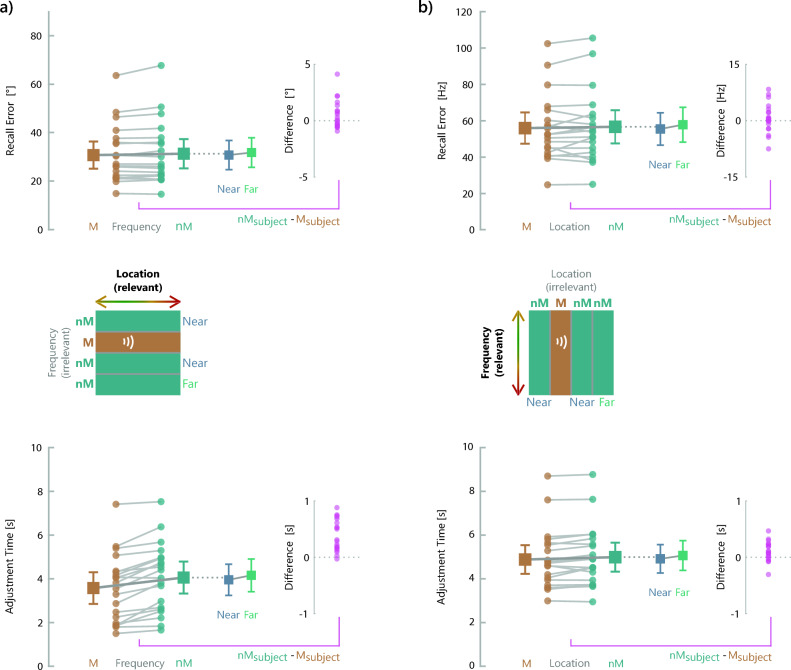
(**a**) Results of Experiment 3 with location as the task-relevant feature and frequency as the irrelevant feature. The upper panel shows precision measured as the location recall error [°] and the lower panel shows adjustment time [s] as a function of variations of the irrelevant feature (frequency). Data for trials with identical values of the irrelevant feature for target and probe stimuli are shown in brown, while trials with a change of the irrelevant feature are shown in turquoise. Colored dots and light gray connecting lines indicate the values of individual participants. Larger squares represent the condition mean with vertical bars indicating the standard error. Smaller squares on the right of each graph show mean and standard error for trials with a smaller difference of the irrelevant feature (“near”, shown in blue) and for trials with a larger difference of the irrelevant feature between sample and test stimuli (“far”, shown in green). The small graphs further on the right show the differences between trials with minus trials without a change of the irrelevant feature. Magenta-colored dots indicate the values of individual participants. (**b**) Results of Experiment 4 with frequency as the task-relevant feature and location as the irrelevant feature.

As expected, recall of the target feature sound location was more accurate when the irrelevant feature frequency was identical to the memorized item compared with when it differed (Fig. 4a, upper panel). The mean recall error amounted to 30.87° (SD = 11.97°) for trials with matching frequency and to 31.43° (SD = 12.86°) for trials with nonmatching frequency (t(19) = 1.98, p_one-tailed_ = 0.031, Cohen’s d = 0.443). The comparison of smaller versus larger differences (“near” versus “far”) of the irrelevant feature between target and test sounds yielded an increased response error for larger than smaller frequency differences. The mean response error was 30.89° (SD = 12.85°) for smaller differences and 31.92° (SD = 12.99°) for larger differences (t(19) = 1.85, p_one-tailed_ = 0.040, Cohen’s d = 0.414).

Correspondingly, adjustment times were longer for nonmatching than for matching values of the irrelevant feature (Fig. 4a, lower panel). The mean adjustment time amounted to 3.60 s (SD = 1.55 s) for matching frequency and to 4.08 s (SD = 1.56 s) for nonmatching frequency (t(19) = 6.15, p_one-tailed_ < 0.001, Cohen’s d = 1.375). Larger frequency differences were accompanied by longer mean adjustment times (4.18 s, SD = 1.60 s) than smaller frequency differences (3.97 s, SD = 1.53 s, t(19) = 3.53, p_one-tailed_ = 0.001, Cohen’s d = 0.79).

### Summary

Experiment 3 showed that changes of the task-irrelevant feature affected working memory performance not only when a match–nonmatch decision was required but also in a continuous recall task that forced subjects to focus all resources on a very precise representation of the relevant feature. The role of the task-irrelevant dimension was further supported by the observation that the irrelevant change effect on both recall error and adjustment time was stronger for larger compared with smaller differences between target and test sounds. The present experiment thus showed that the irrelevant change effect in AWM is not specific to match–nonmatch tasks but generalizes to continuous recall tasks.

## Experiment 4

By reversing the task relevance of the feature dimensions location and frequency compared with Experiment 3, this experiment assessed the effects of variations of the irrelevant dimension location on memory performance for sound frequency.

### Methods

#### Participants

Twenty healthy young adults (16 females, mean age 23.15, SD = 3.28 years) participated in Experiment 4. None of the participants had taken part in any of the previous experiments. They fulfilled the same criteria as in Experiments 1–3. Written informed consent was obtained from all observers. They were naive to the purpose of the experiment and received a remuneration of €12 per hour.

#### Stimuli

The same sample stimuli were used as in Experiments 1–3. The frequency of the test sounds was drawn randomly from the range of 246.942–783.99 Hz. In contrast, the location of the test sound matched the target location in half of the trials, whereas in the other half of trials, it was presented at one of the two locations that had not been presented in the trial yet.

#### Procedure

The experimental settings and the procedure were almost identical to Experiment 3. The main difference was that frequency was the task-relevant and location the task-irrelevant feature. As in Experiment 3, participants had to perform a continuous recall task. After the delay phase, a 500-m probe tone was presented and continuously repeated either until a response was given or the 20-s response window ended. The probe tone had a random start value on the to-be-reported feature dimension (frequency). On the task-irrelevant feature dimension, it was either identical to or differed from the target stimulus in half of the trials each. Participants adjusted the target feature frequency by moving a computer mouse to the left or right and entered their response via left mouse button press. The continuous response space spanned the frequency range from 246.942 to 783.99 Hz. Participants received feedback about their recall precision at the end of each trial as described above for Experiment 3.

#### Data analysis

The data analysis followed the same principles as in Experiment 3.

### Results

When participants actively recalled the target feature frequency, there was no effect of the irrelevant feature on recall error (Fig. 4b, upper panel). The mean response error amounted to 55.96 Hz (SD = 18.34 Hz) for trials with matching location and to 56.67 Hz (SD = 19.44 Hz) for trials with nonmatching location (t(19) = 0.819, p_one-tailed_ = 0.212, Cohen’s d = 0.183). However, comparing smaller with larger differences (“near” versus “far”) on the irrelevant feature location between target and test sounds yielded the expected increased response error for larger than smaller differences. The mean response error amounted to 55.49 Hz (SD = 18.98 Hz) for smaller differences and 57.82 Hz (SD = 20.39 Hz) for larger differences, t(19) = 1.82, p_one-tailed_ = 0.043, Cohen’s d = 0.406.

Adjustment times were longer for nonmatching than for matching values of the irrelevant feature location (Fig. 4b, lower panel). The mean adjustment time amounted to 4.90 s (SD = 1.41 s) for matching location and to 5.01 s (SD = 1.42 s) for nonmatching location (t(19) = 2.80, p_one-tailed_ = 0.006, Cohen’s d = 0.626). Similarly to Experiment 3, the distance between target and test stimuli on the irrelevant feature affected adjustment times. They were shorter for smaller (4.94 s, SD = 1.40 s) than larger location differences (5.09 s, SD = 1.45 s, t(19) = 2.26, p_one-tailed_ = 0.018, Cohen’s d = 0.506).

### Summary

Experiment 4 showed that changes of task-irrelevant location affected adjustment time but not recall precision for memorized sound frequency. Participants took longer to enter their response when sound location differed between target and test stimuli than when it remained unchanged. This means that changes of task-irrelevant location made it more difficult to access the relevant feature, whereas they did not reduce the accuracy of its recall from memory. In addition, adjustment times were longer for larger differences in location than for smaller differences. While response errors did not differ between trials with and without changes of the irrelevant feature, responses were less accurate for larger compared with smaller location differences between target and test stimuli. This suggests that an irrelevant change effect on response error existed for larger location differences.

### Discussion

The present study used the irrelevant change effect to investigate object-based attention in AWM. We found evidence for the memory processing of task-irrelevant sound features across a series of four experiments varying in terms of both irrelevant feature (sound frequency or perceived location) and response mode (match–nonmatch task or continuous recall). In the two match–nonmatch experiments, changes of the irrelevant feature affected both correct response rate and reaction time. Matches of the relevant feature were detected more accurately and faster when target and probe matched also on the irrelevant feature. While this replicates previous reports^23,24^, the present study also showed a pronounced irrelevant change effect for nonmatch trials. Here mismatch detection was facilitated when there was also a change of the irrelevant feature. These effects were more pronounced for larger than smaller differences of the irrelevant feature, providing indirect evidence suggesting that a fairly detailed representation of the irrelevant information was represented in AWM. Signal detection analysis showed that changes of task-irrelevant frequency reduced the sensitivity of location recall, whereas there was merely a statistical trend for an effect of irrelevant location changes on the sensitivity of frequency recall. Effects on the criterion were observed for both irrelevant frequency and location. There was a bias towards a ‘match’ response when the irrelevant feature also matched between sample and probe. Apparently, the match of the irrelevant feature led to a strong tendency to judge the entire sound object (including the relevant feature) as matching. In contrast, for nonmatches of the irrelevant feature, participants were not biased towards either ‘match’ or ‘nonmatch’ responses.

Irrelevant feature effects did not only occur in tasks requiring match–nonmatch comparisons, but also in continuous recall tasks in our third and fourth experiments. For location, recall error showed a small increase for changes of irrelevant frequency. In contrast, there was no effect of irrelevant location change on frequency recall. In both continuous recall experiments, the comparison between smaller versus larger changes of the irrelevant feature revealed that performance differences scaled with the magnitude of irrelevant feature change. The continuous report procedure aims at measuring the quality of memory representations rather than the speed at which they are accessed. Adjustment times are therefore less informative about memory performance than accuracy. However, these two variables are related to each other and may underlie a similar speed-accuracy tradeoff as correct responses and reaction times in a change detection task. Accordingly, we did find consistent effects of irrelevant feature changes across both continuous report experiments with longer adjustment times for irrelevant feature nonmatches for both irrelevant frequency and location.

Comparing match–nonmatch with active recall task paradigms, we found consistent effects of irrelevant feature changes on all dependent measures in both tasks when location was task-relevant and frequency irrelevant. For task-relevant frequency and irrelevant location, we found consistent effects on reaction/adjustment times. For d′ (mere statistical trend) and recall error (no effect), the absence of effects was also consistent between tasks.

How do these findings contribute to our knowledge about object representations in AWM? AWM enables following auditory objects over time, requiring some means of matching their serial occurrences to decide whether the current object still represents the same or a novel object. This likely relies on matching occurrences according to their similarity with respect to their most relevant (target) feature. However, in situations with multiple objects characterized by overlapping features, focusing on a single feature might not be sufficient. Instead, object stability may rely on the complete machinery that codes object identity by incorporating all available accompanying features including serial and spatial position, that might appear irrelevant but do support object discrimination. In the visual domain, we have shown that object stability (serial dependence) is supported by accompanying context features. We found that spatial position still affected serial dependence, even if it was completely task-irrelevant^29^. This indicated that it was automatically integrated into the visual object representation. The present findings suggest that both frequency and location may play similar roles for auditory objects.

However, we did not test memory for the irrelevant feature explicitly, for example in surprise trials^30^, as this would have violated our principle of keeping one feature completely task-irrelevant throughout an entire experiment. In summary, our results are consistent with the assumption that sounds are stored in AWM as auditory objects^17,31^. Thus object-based attention can lead to an involuntary maintenance of task-irrelevant features similar to the visual modality^20,21^.

Concerning possible differences between frequency and location as irrelevant features, previous studies have found clear differences between stronger effects of irrelevant auditory ‘pattern’ features like sound content, pitch, or timbre and weaker effects (or even an absence of effects) of irrelevant location^23,24^. The results pattern was less clear in our study. While changes of irrelevant frequency affected all dependent variables, effects of irrelevant location change were observed for accuracy in Experiment 2 and for reaction/adjustment times in both Experiments 2 and 4, but they did not reach significance for sensitivity (d′) in Experiment 2 or recall error in Experiment 4. While there seemed to be a trend for a more pronounced effect of irrelevant frequency than location as in the previous studies, based on this mixed set of results, the evidence seems insufficient to make a strong claim about increased incidental binding of frequency compared with location.

In contrast to previous studies where task relevance varied between features in the course of an experiment^24^, we ensured that the irrelevant feature remained strictly irrelevant throughout the entire experiment. In addition, we recruited non-overlapping groups of participants, so that a possible spillover of processing strategies from one experiment to the next could be excluded. In addition, the present study used a retro-cue paradigm with two sample stimuli that had to be encoded into AWM before one of them was cued for further maintenance. This allowed to test whether effects of the putatively bound irrelevant feature would be detectable even when participants could clearly focus on only one feature of a single stimulus. Besides, this trial structure was consistent with current neuroimaging studies on AWM^25–27^. As the irrelevant feature always differed between the two sample sounds, it might have supported their encoding as distinct objects and the retrospective attentional selection of the cued item in AWM. The extent to which this possible function of the irrelevant feature has contributed to the observed effects could be tested by using either one sample sound only or by choosing identical values for the irrelevant feature of both sample stimuli.

Our results suggest that the effects consisted of a combination of response bias and quality of the memory representation. First, match vs. nonmatch of the irrelevant feature influenced the memory-based decision. This was observed consistently for both frequency and location, showing that information about the irrelevant feature was not only encoded, but also retained in working memory although it could have been deleted. Second, changes of irrelevant frequency but not location also led to a poorer memory representation, as reflected by both reduced sensitivity and recall accuracy. While previous studies have focused mainly on response accuracy^23,24^, the present study provides novel evidence concerning memory-based decision making.

Previous studies on visual short-term memory have suggested that irrelevant feature processing was transient^10^ and that sustained maintenance across a longer delay phase depended on voluntary effort^11,32^. For example, Logie et al.^22^ found the irrelevant change effect to disappear at delays of > 1500 ms, which was interpreted as a consequence of control processes selectively inhibiting the irrelevant information. Others have reported that a visual mask abolished the irrelevant-change effect, suggesting that it was attributable to a sensory memory representation^33^. While it is difficult to compare the temporal dynamics between visual and auditory working memory, the present findings are inconsistent with an attribution of the irrelevant feature effect to sensory memory. In line with a recent visual working memory study that did not find backward masking to abolish the irrelevant change effect, thus excluding sensory memory as an explanation^34^, our retro-cue paradigm made it very unlikely that the task could have been solved on the basis of sensory memory traces. Furthermore, it has been suggested that irrelevant features are encoded under conditions of low memory load only^10^. As we did not manipulate load systematically, we cannot exclude that the irrelevant change effects may have been abolished by higher memory load. In summary, the present findings suggest that at a moderate task difficulty, participants are not able to ignore the irrelevant feature completely across a delay duration of 3 s. This was true for task-irrelevant sound frequency, while the effects were somewhat less pronounced for location.

What are the neural correlates of irrelevant feature processing? Applying multivariate pattern analysis (MVPA) to functional magnetic resonance imaging (fMRI) data during a task requiring to maintain either stimulus color or orientation in visual working memory revealed that classification was successful only for the actively maintained feature^35^. A more recent electroencephalography (EEG) study using MVPA found that task-irrelevant features could be decoded only during the encoding phase but not during the delay period of a working memory task^36^. However, the decoding of task-irrelevant information seems to depend on the type of feature. Foster et al.^37^ decoded task-irrelevant spatial representations from EEG alpha activity during the retention phase of a visual working memory task. Whether the same might be true for task-irrelevant frequency and location in AWM is a matter of future research.

In conclusion, the present series of AWM experiments found that changes of task-irrelevant features between encoding and test affected performance for the task-relevant feature. The strength of this irrelevant change effect scaled with the magnitude of change and was present for both match–nonmatch and continuous recall tasks. Our results suggest that object-based attention in AWM leads to an involuntary maintenance of task-irrelevant sound features.

## Supplementary Information

Supplementary Tables.

## Data Availability

The datasets generated during and/or analyzed during the current study are available from the corresponding author on reasonable request.
