# Supplementary material for: Auditory objects in working memory include task-irrelevant features
Source: Sci Rep. 2024 Sep 11;14:21216. doi: 10.1038/s41598-024-72177-6 (PMC11390711; doi:10.1038/s41598-024-72177-6)
Supplement: Supplementary file 1 — Supplementary Tables. [file 41598_2024_72177_MOESM1_ESM.docx]

**Auditory objects in working memory include task-irrelevant features**

**Supplementary Information**

**Experiment 1**

**Supplementary Table S1. ANOVA results for Experiment 1.**

| **Dependent variable** | F(1,19) | p-value | effect size ($\eta_{p}^{2})$ |
| --- | --- | --- | --- |
| **Accuracy** |  |  |  |
| Main effect Location Variation | 13.00 | 0.002 | 0.406 |
| Main effect Frequency Variation | 9.84 | 0.005 | 0.341 |
| Interaction | 30.65 | < 0.001 | 0.617 |
| **Accuracy ("near" vs. "far" comparison)** |  |  |  |
| Main effect Location Variation | 0.09 | 0.768 |  |
| Main effect Frequency Change Magnitude | 1.28 | 0.272 |  |
| Interaction | 13.71 | 0.002 | 0.419 |
| **Reaction time** |  |  |  |
| Main effect Location Variation | 1.15 | 0.297 |  |
| Main effect Frequency Variation | 25.02 | < 0.001 | 0.568 |
| Interaction | 17.95 | < 0.001 | 0.486 |
| **Reaction time ("near" vs. "far" comparison)** |  |  |  |
| Main effect Location Variation | 3.36 | 0.083 |  |
| Main effect Frequency Change Magnitude | 0.17 | 0.685 |  |
| Interaction | 0.63 | 0.436 |  |

Note. Effect sizes were calculated for significant effects only. For the "near" vs. "far" comparisons, only those trials with a change of the irrelevant feature frequency (half of the total number of trials) were entered into the analysis. The factor Frequency Change Magnitude had two factors ("near" vs. "far").

**Supplementary Table S2. Accuracy (% correct) and reaction times (ms) for Experiment 1.**

|  |  | **Accuracy**  **Frequency** | | | |  | **Reaction time**  **Frequency** | | | |
| --- | --- | --- | --- | --- | --- | --- | --- | --- | --- | --- |
|  |  | match | | nonmatch | |  | match | | nonmatch | |
| **Location** |  | mean | SD | mean | SD |  | mean | SD | mean | SD |
| match |  | 83.7 | 14.2 | 68.2 | 14.6 |  | 260 | 129 | 346 | 128 |
| nonmatch |  | 59.5 | 16.9 | 69.1 | 14.2 |  | 314 | 134 | 318 | 141 |

**Supplementary Table S3. Accuracy (% correct) for smaller versus larger changes ("near" versus "far", respectively) of the task-irrelevant feature Frequency in Experiment 1.**

|  |  | **Accuracy**  **Frequency change** | | | |
| --- | --- | --- | --- | --- | --- |
|  |  | near | | far | |
| **Location** |  | mean | SD | mean | SD |
| match |  | 71.8 | 15.8 | 64.5 | 14.7 |
| nonmatch |  | 67.5 | 15.2 | 71.0 | 14.8 |

**Experiment 2**

**Supplementary Table S4. ANOVA results for Experiment 2.**

| **Dependent variable** | F(1,19) | p-value | effect size ($\eta_{p}^{2})$ |
| --- | --- | --- | --- |
| **Accuracy** |  |  |  |
| Main effect Frequency Variation | 19.49 | < 0.001 | 0.506 |
| Main effect Location Variation | 0.75 | 0.396 |  |
| Interaction | 53.85 | < 0.001 | 0.739 |
| **Accuracy ("near" vs. "far" comparison)** |  |  |  |
| Main effect Frequency Variation | 2.60 | 0.123 |  |
| Main effect Location Change Magnitude | 1.50 | 0.236 |  |
| Interaction | 57.07 | < 0.001 | 0.750 |
| **Reaction time** |  |  |  |
| Main effect Frequency Variation | 0.56 | 0.462 |  |
| Main effect Location Variation | 0.12 | 0.730 |  |
| Interaction | 15.32 | < 0.001 | 0.446 |
| **Reaction time ("near" vs. "far" comparison)** |  |  |  |
| Main effect Frequency Variation | 0.64 | 0.434 |  |
| Main effect Location Change Magnitude | 7.87 | 0.011 | 0.293 |
| Interaction | 16.53 | < 0.001 | 0.465 |

Note. Effect sizes were calculated for significant effects only. For the "near" vs. "far" comparisons, only those trials with a change of the irrelevant feature location (half of the total number of trials) were entered into the analysis. The factor Location Change Magnitude had two factors ("near" vs. "far").

**Supplementary Table S5. Accuracy (% correct) and reaction times (ms) for Experiment 2.**

|  |  | **Accuracy**  **Location** | | | |  | **Reaction time**  **Location** | | | |
| --- | --- | --- | --- | --- | --- | --- | --- | --- | --- | --- |
|  |  | match | | nonmatch | |  | match | | nonmatch | |
| **Frequency** |  | mean | SD | mean | SD |  | mean | SD | mean | SD |
| match |  | 81.6 | 8.4 | 73.1 | 8.2 |  | 270 | 105 | 289 | 112 |
| nonmatch |  | 61.1 | 15.3 | 68.3 | 14.6 |  | 301 | 124 | 279 | 112 |

**Supplementary Table S6. Accuracy (% correct) and reaction times (ms) for smaller versus larger changes ("near" versus "far", respectively) of the task-irrelevant feature Location in Experiment 2.**

|  |  | **Accuracy**  **Location change** | | | |
| --- | --- | --- | --- | --- | --- |
|  |  | near | | far | |
| **Frequency** |  | mean | SD | mean | SD |
| match |  | 77.3 | 8.6 | 69.2 | 9.2 |
| nonmatch |  | 65.6 | 14.1 | 71.0 | 15.5 |
